# The Prognostic Value of CD133 in Predicting the Relapse and Recurrence Pattern of High-Grade Gliomas on MRI: A Meta-Analysis

**DOI:** 10.3389/fonc.2021.722833

**Published:** 2021-09-02

**Authors:** Mahdi Abdoli Shadbad, Negar Hosseinkhani, Zahra Asadzadeh, Oronzo Brunetti, Nicola Silvestris, Behzad Baradaran

**Affiliations:** ^1^Student Research Committee, Tabriz University of Medical Sciences, Tabriz, Iran; ^2^Immunology Research Center, Tabriz University of Medical Sciences, Tabriz, Iran; ^3^Medical Oncology Unit, IRCCS IstitutoTumori “Giovanni Paolo II” of Bari, Bari, Italy; ^4^Department of Biomedical Sciences and Human Oncology, University of Bari “Aldo Moro”, Bari, Italy; ^5^Department of Immunology, Tabriz University of Medical Sciences, Tabriz, Iran; ^6^Pharmaceutical Analysis Research Center, Tabriz University of Medical Sciences, Tabriz, Iran

**Keywords:** CD133, high-grade gliomas, high-grade gliomas relapse, high-grade gliomas recurrence pattern, progression-free survival, time to local recurrence, time to distant recurrence, magnetic resonance imaging

## Abstract

**Background:**

Cancer stem cells have been implicated in tumor relapse, tumor invasion, and cancer therapy resistance in high-grade gliomas; thus, characterizing cancer stem cell-related markers can help determine the prognosis of affected patients. Preclinical studies have reported that CD133 is implicated in tumor recurrence and cancer therapy resistance in high-grade gliomas; however, clinical studies have reported inconclusive results regarding its prognostic value in patients with high-grade gliomas.

**Methods:**

We systematically searched the PubMed, Scopus, Web of Science, and Embase databases to obtain peer-reviewed studies published before March 10, 2021. Then, we conducted the current systematic review and meta-analysis based on the preferred reporting items for systematic reviews and meta-analyses (PRISMA) statements. By applying the random-effect model, the effect size of studies investigating the progression-free survival (PFS), time to local recurrence (TTL), and time to distant recurrence (TTD) were calculated using RevMan version 5.4. The heterogeneity between the included studies was studied by the I^2^ index and Cochran’s Q test. Egger test was performed on funnel plots to investigate the potential asymmetry and publication bias among the included studies using CMA version 2.

**Results:**

With the 10% cut-off, CD133 protein overexpression is associated with the inferior PFS of patients with high-grade gliomas. Increased CD133 protein expression is associated with sooner distant tumor recurrence on MRI in glioblastoma patients and patients with high-grade gliomas and improved TTL on MRI in glioblastoma patients.

**Conclusion:**

Based on the current evidence from 1086 patients with high-grade gliomas, CD133 overexpression is a valuable marker to predict tumor relapse and tumor recurrence patterns in patients with high-grade gliomas.

## Introduction

Gliomas are among the frequently diagnosed primary brain tumors; however, the prognosis of affected patients is not favorable. Indeed, tumor recurrence and cancer therapy resistance have posed daunting challenges for patients with high-grade gliomas (1). Therefore, a better understanding of the biology of high-grade gliomas might pave the way for introducing novel biomarkers that can predict the prognosis of affected patients.

Cancer stem cells are a small population of tumor bulk that has been introduced as the main culprit of tumor relapse. The stemness and self-renewal features of these tumoral cells can give rise to a malignant tumor after the initial therapy. Besides tumor development, cancer stem cells can facilitate tumor migration (2–4). These tumor-initiating cells have also been implicated in chemoresistance. Indeed, the overexpression of aldehyde dehydrogenase-I, stimulated DNA repair mechanisms, reduced chemotherapeutic agents influx, and increased their efflux are among the proposed mechanisms for cancer stem cells-mediated chemoresistance in high-grade gliomas (5). It has been reported that these cells can stimulate the Wnt/β-catenin pathway, leading to stemness, increased invasion, and proliferation in high-grade gliomas (6, 7). Wickström et al. have indicated that the stimulation of the Wnt/β-catenin pathway is highly associated with temozolomide resistance in glioblastoma *via* activating MGMT (8).

Preclinical findings have indicated that CD133 is overexpressed in cancer stem cells of high-grade gliomas. It has been shown that CD133 can activate the Wnt/β-catenin pathway, maintaining the cancer stem cell population in glioblastoma. Indeed, the expression level of Wnt/β-catenin-related signals in CD133-positive cancer stem cells has been substantially increased compared to differentiated CD133-negative glioblastoma cells (9). Growing evidence indicates that CD133 can increase proliferation, induce cancer therapy resistance, and maintain stemness in glioblastoma (10–13). Despite the critical roles of CD133 in tumor development, there is a controversy about the prognostic value of CD133 in predicting tumor relapse and its recurrence pattern in high-grade gliomas (14–17).

Herein, the current study aims to determine the prognostic value of CD133 in predicting tumor relapse and tumor recurrence patterns in patients with high-grade gliomas. The results of this current study can be translated into clinical practice to predict tumor recurrence and its patterns in patients with high-grade gliomas.

## Methods

This study was performed according to the PRISMA statements (18).

### Search Strategy

The PubMed, Scopus, Web of Science, and Embase databases were systematically searched to obtain peer-reviewed records published before March 10, 2021, with the following keywords: (“CD133” OR “prominin-1” OR “AC133” OR “AC133 antigen” OR “PROML1” OR “AC133 antigen” OR “prominin-like protein 1” OR “CORD12” OR “RP41” OR “MSTP061” OR “MCDR2” OR “STGD4” OR “prominin 1” OR “prominin (mouse)-like 1” OR “HProminin” OR “PROM1” OR “antigen AC133” OR “CD133 antigen”) and (“glioblastoma” OR “glioblastoma multiforme” OR “high-grade glioma” OR “high grade glioma” OR “anaplastic” OR “anaplastic astrocytoma” OR “astrocytoma” OR “grade III glioma” OR “grade IV glioma”) and (“local recurrence” OR “distant recurrence” OR “local relapse” OR “distant relapse” OR “local recurrent” OR “distant recurrent” OR “local-recurrence” OR “distant-recurrence” OR “local-relapse” OR “distant-relapse” OR “local-recurrent” OR “distant-recurrent” OR “LRFS” OR “local recurrence-free survival” OR “distant recurrence-free survival” OR “DRFS” OR “local recurrence free survival” OR “distant recurrence free survival” OR “local-recurrence-free survival” OR “distant-recurrence-free survival” OR “DRFS” OR “local-recurrence free survival” OR “distant-recurrence free survival” OR “relapse-free survival” OR “recurrence-free survival” OR “relapse free survival” OR “recurrence free survival” OR “RFS” OR “local relapse-free survival” OR “local relapse free survival” OR “local-relapse-free survival” OR “local-relapse free survival” OR “distant relapse free survival” OR “distant relapse-free survival” OR “distant-relapse-free survival” OR “distant-relapse free survival” OR “time to progression” OR “time-to-progression” OR “time to-progression” OR “time-to progression” OR “TTP” OR “progression-free survival” OR “PFS” OR “progression free survival” OR “disease free survival” OR “disease-free survival” OR “DFS” OR “distant recurrence-free interval” OR “distant recurrence free interval” OR “distant-recurrence free interval” OR “distant-recurrence-free interval” OR “recurrence-free interval” OR “recurrence free interval” OR “DDFS” OR “distant disease-free survival” OR “distant disease free survival” OR “distant-disease free survival” OR “distant-disease-free survival” OR “IDFS” OR “invasive disease-free survival” OR “invasive disease free survival” OR “invasive-disease free survival” OR “invasive-disease-free survival” OR “prognosis” OR “prognostic” OR “prognoses”). We incorporated the Emtree and MeSH terms to increase the sensitivity of our systematic search.

### Definition of Recurrence Pattern Indices

To investigate the prognostic value of CD133 expression in determining relapse and recurrence patterns of high-grade gliomas, we investigated the association between the protein expression of CD133 with TTL, TTD, and PFS. Local recurrence is defined as a new enhanced lesion on MRI contiguous (< 3cm) the resection site, and distant recurrence is defined as a new enhanced lesion on MRI away (> 3cm) from the resection site (16, 17, 19).

### Study Selection and Data Extraction

After the systematic search, the retrieved studies were screened in two phases. In the first phase, the obtained papers were independently reviewed by two authors (M.A.S and N.H) based on their titles and abstracts. In the second phase, the full text of the records and their supplementary data were independently reviewed by the same authors for consideration to be included in the current meta-analysis. Any disagreements were resolved *via* consulting with B.B and consensus.

### The Inclusion and the Exclusion Criteria

Studies with the following eligibility criteria were included in the current meta-analysis: (1) clinical studies, (2) investigations with the objectives of studying the association between CD133 expression with the relapse and the recurrence pattern-related survival indices, i.e., TTL, TTD, and PFS, in patients with high-grade gliomas, (3) studies that investigated the protein expression of CD133 in high-grade gliomas, and (4) studies that were published in English.

Records with the following criteria were excluded from the meta-analysis: (1) studies that failed to fulfill the inclusion criteria as mentioned above, (2) studies that only investigated the mRNA expression of CD133, rather than protein expression of CD133, (3) investigations that did not provide the prognostic data regarding the CD133 expression, (4) meeting abstracts, (5) duplicated records, (6) book chapters, (7) preclinical studies, (8) review articles, and (9) studies that were solely based on bioinformatics.

### Data Extraction

The following data were extracted from the included studies: (1) the first author and the year of publication, (2) the sample size, (3) the country, (4) the glioma grade, (5) the measured end-point, (6) the detection method, (7) cut-off for considering CD133 expression as high, and (8) the HR and the 95% confidence interval (CI) of measured PFS, TTD, and TTL.

### Evaluating the Quality of Included Studies

We assessed the quality of the included studies to facilitate the translation of our obtained results into routine practice. The quality of included studies has been evaluated according to the Hayden et al. checklist (20).

### Statistical Analysis

We used RevMan version 5.4 to conduct our meta-analyses. The common effect sizes were calculated as HR to assess the association between the protein expression of CD133 with the relapse and the recurrence pattern of high-grade gliomas in affected patients. Since there might be unpublished investigations regarding this topic, we applied the random-effect model. Like our previous investigation, the standard chi-squared test (Cochran Q test) and I^2^ index were used to assess the possible heterogeneity among the included studies (21). The values over 50% for the I^2^ index were considered as high heterogeneity. To visualize the potential asymmetry and publication bias, we provided funnel plots using CMA version 2. Besides, we performed the Egger test to evaluate the potential publication bias in an objective manner.

## Results

### The Results of the Systematic Review

**Figure 1:**
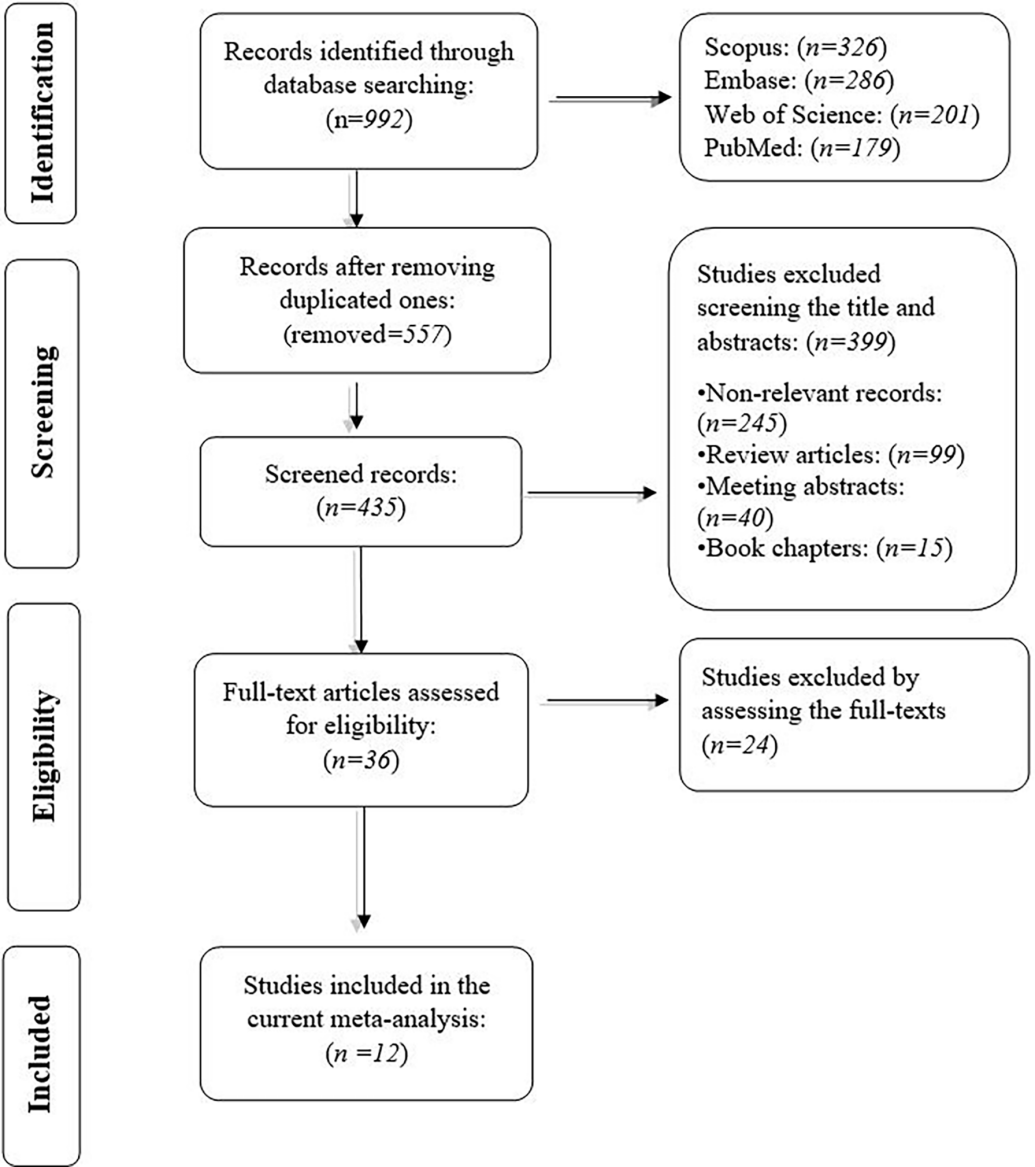
The flowchart of literature identification, inclusion, and exclusion in the current systematic review.

We obtained 992 records from PubMed (*n*=179), and Web of Science (*n*=201), Embase (*n*=286), and Scopus (*n*=326). After removing duplicated studies, the title and the abstracts of the remaining studies were screened. In the second phase, the full text of the remaining 36 studies and their supplementary data were reviewed for consideration to be included in the current meta-analysis. Following the exclusion of the 24 studies, we extracted the data from the remaining 12 studies. The flowchart of our systematic review process is demonstrated in **Figure 1**. The characteristics of the included studies are shown in **Table 1**.

**Table 1:** The characteristics of the twelve included studies.

No.	First author, year	Country	Sample size	Glioma grade	Endpoint	Detection method	Cut-off
1	Tetsu Yamaki, 2020 (17)	Japan	167	IV	TTD and TTL	The integration of IHC with Western blot	Ratio>1
2	Yasuo Iwadate, 2017 (15)	Japan	70	IV	PFS	IHC	10%
3	Yasuo Iwadate, 2016 (14)	Japan	80	IV	PFS	IHC	10%
4	Ichiyo Shibahara, 2015 (16)	Japan	86	III	TTD and TTL	The integration of IHC with Western blot	Ratio > 1
5	Rikke H Dahlrot, 2014 (22)	Denmark	211	III and IV	PFS	Immunofluorescence	2%
6	Jung Ha Shin, 2013 (23)	South Korea	67	IV	PFS	IHC	50%
7	Ichiyo Shibahara, 2013 (19)	Japan	112	IV	TTD and TTL	The integration of IHC with Western blot	Ratio > 1
8	Consolación Melguizo, 2012 (24)	Spain and Italy	78	IV	PFS	IHC	25%
9	Kyung-Jung Kim, 2011 (25)	South Korea	88	IV	PFS	IHC	50%
10	JIE HE, 2011 (26)	China	59	III and IV	PFS	IHC	10%
11	Roberto Pallini, 2008 (27)	Italy	44	IV	PFS	Immunofluorescence	2%
12	Felix Zeppernick, 2008 (28)	Germany	24	III	PFS	IHC	1%

TTD, time to distant recurrence; TTL, time to local recurrence; PFS, progression-free survival, and IHC, immunohistochemistry.

The included studies have been published between 2008 to 2020. The included studies concerning the PFS of affected patients have applied different cut-offs for considering protein CD133 expression as high. However, the studies investigating CD133 protein expression with TTL and TTD have applied a unified method and cut-off (**Table 1**).

### The Protein Expression of CD133 and PFS of Patients With High-Grade Gliomas

**Figure 2:**
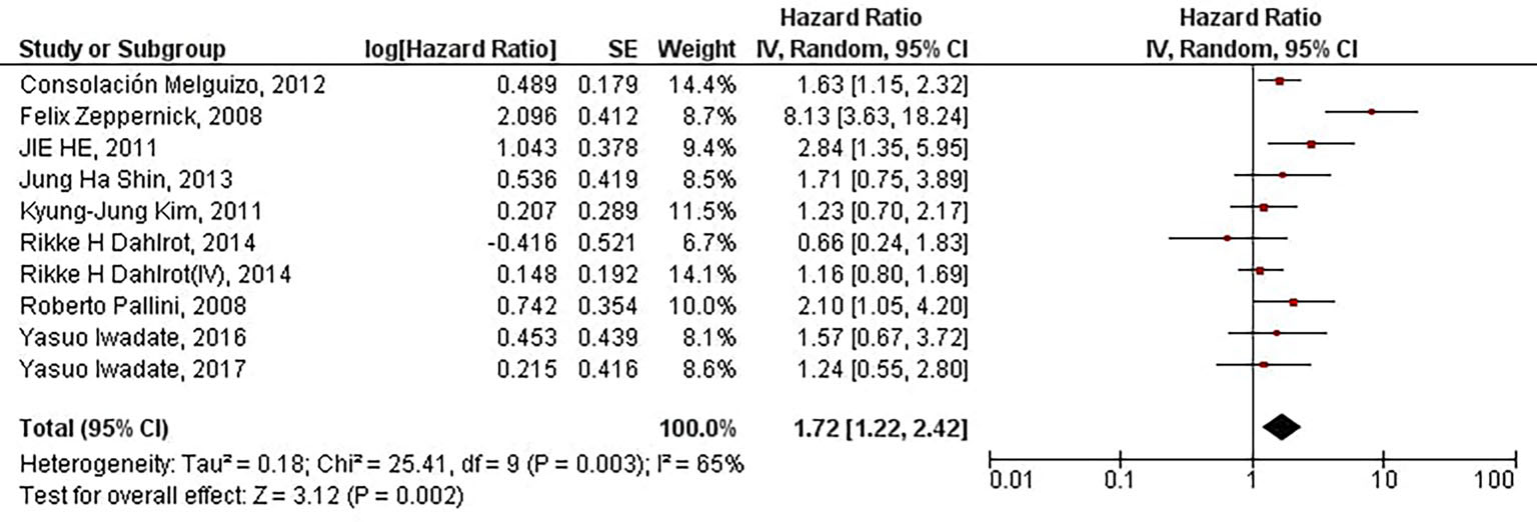
The forest plot of studies evaluating the prognostic value of CD133 overexpression in determining the PFS of patients with high-grade gliomas; Rikke H Dahlrot (IV) pertains to the Dahlrot et al.’s study on patients with grade IV gliomas, and Rikke H Dahlrot pertains to the Dahlrot et al.’s study on patients with grade III gliomas.

Our results have indicated that increased protein expression of CD133 is significantly associated with the inferior PFS of patients with high-grade gliomas (HR = 1.72, 95% CI: 1.22 – 2.42, P = 0.002). Our results have also indicated a high and significant heterogeneity between the included studies (I^2^ = 65%, P = 0.003) (**Figure 2**). Therefore, the meta-analyses of the included studies based on CD133 protein overexpression cut-offs have been conducted to address the high and significant heterogeneity.

#### The Protein Expression of CD133 and PFS of Patients With High-Grade Gliomas With 2% Cut-Off

**Figure 3:**
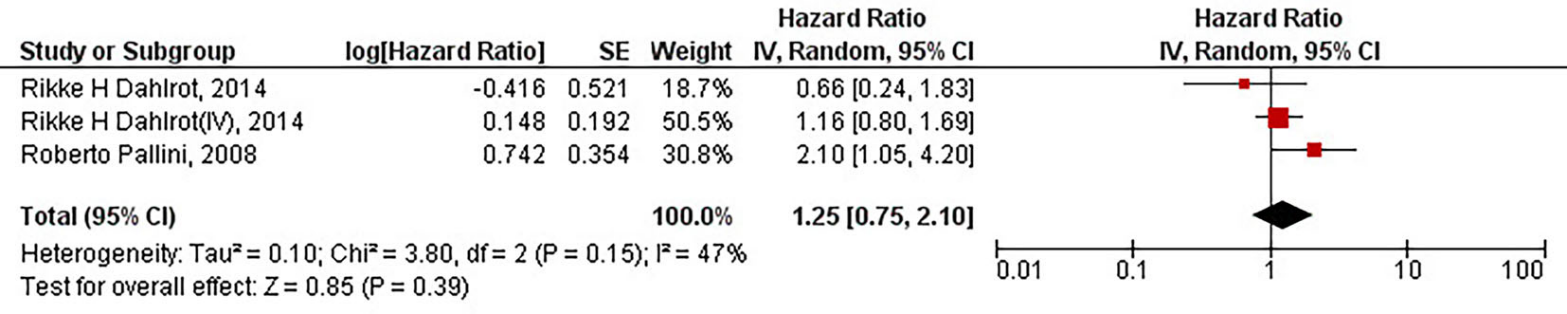
The forest plot of studies evaluating the prognostic value of CD133 overexpression in determining the PFS of patients with high-grade gliomas (with 2% cut-off); Rikke H Dahlrot (IV) pertains to the Dahlrot et al.’s study on patients with grade IV gliomas, and Rikke H Dahlrot pertains to the Dahlrot et al.’s study on patients with grade III gliomas.

Our results have demonstrated that the protein overexpression of CD133 with 2% cut-off is not significantly associated with the inferior PFS of patients with high-grade gliomas (HR = 1.25, 95% CI: 0.75 – 2.10, P = 0.39). Our results have also indicated no significant heterogeneity between the included studies (I^2^ = 47%, P = 0.15) (**Figure 3**).

#### The Protein Expression of CD133 and PFS of Patients With High-Grade Gliomas With 10% Cut-Off

**Figure 4:**
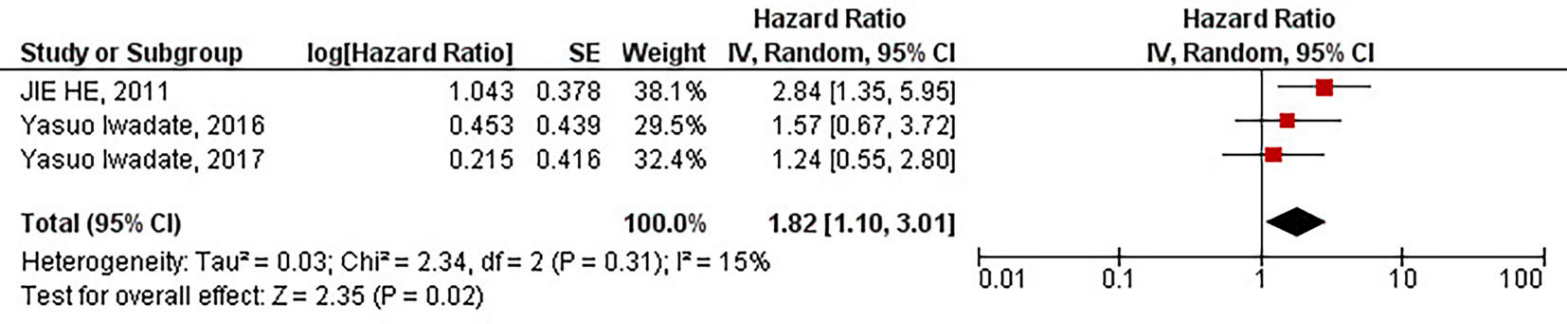
The forest plot of studies evaluating the prognostic value of CD133 overexpression in determining the PFS of patients with high-grade gliomas (with 10% cut-off).

Our results have demonstrated that the protein overexpression of CD133 with 10% cut-off is significantly associated with the inferior PFS of patients with high-grade gliomas (HR = 1.82, 95% CI: 1.10 – 3.01, P = 0.02). Besides, our results have shown no significant heterogeneity between the included studies (I^2^ = 15%, P = 0.31) (**Figure 4**).

#### The Protein Expression of CD133 and PFS of Patients With High-Grade Gliomas With 50% Cut-Off

**Figure 5:**
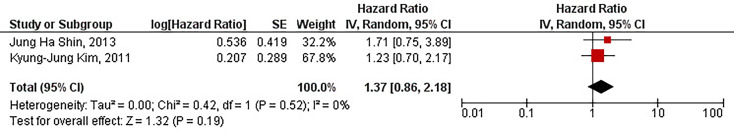
The forest plot of studies evaluating the prognostic value of CD133 overexpression in determining the PFS of patients with high-grade gliomas (with 50% cut-off).

Our study has shown that the protein overexpression of CD133 with 50% cut-off is not significantly associated with the inferior PFS of patients with high-grade gliomas (HR = 1.37, 95% CI: 0.86 – 2.18, P = 0.19). Our results have also shown no significant heterogeneity between the included studies (I^2^ = 0%, P = 0.52) (**Figure 5**).

### The Protein Expression of CD133 and TTD of Glioblastoma Patients

**Figure 6:**
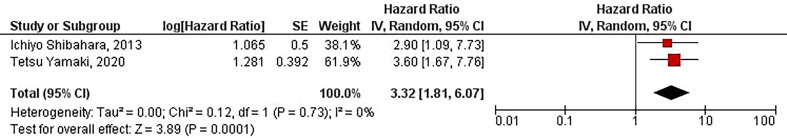
The forest plot of studies evaluating the prognostic value of CD133 in determining the TTD of glioblastoma patients.

The current study has shown that increased protein expression of CD133 is significantly associated with sooner distant recurrence of gliomas in glioblastoma patients (HR = 3.32, 95% CI: 1.81 – 6.07, P = 0.0001). Besides, we have found no significant heterogeneity between the included studies (I^2^ = 0%, P = 0.73) (**Figure 6**).

### The Protein Expression of CD133 and TTD of Patients With High-Grade Gliomas

**Figure 7:**
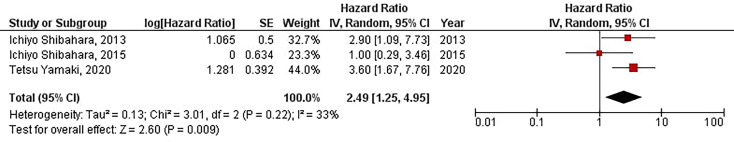
The forest plot of studies evaluating the prognostic value of CD133 in determining the TTD of patients with high-grade gliomas.

Our results have demonstrated that increased protein expression of CD133 is significantly associated with a shorter time of distant tumor recurrence in patients with high-grade gliomas (HR = 2.49, 95% CI: 1.25 – 4.95, P = 0.009). Besides, we have found no significant heterogeneity between the included studies (I^2^ = 33%, P = 0.22) (**Figure 7**).

### The Protein Expression of CD133 and TTL of Glioblastoma Patients

**Figure 8:**
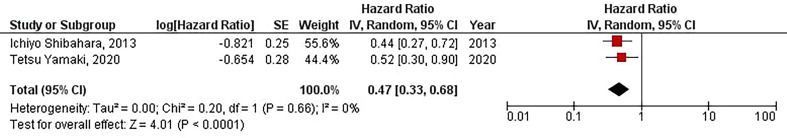
The forest plot of studies evaluating the prognostic value of CD133 in determining the TTL of glioblastoma patients.

The current meta-analysis has indicated that elevated protein expression of CD133 is significantly associated with improved TTL in glioblastoma patients (HR = 0.47, 95% CI: 0.33 – 0.68, P < 0.0001). Besides, we have found no significant heterogeneity between the included studies (I^2^ = 0%, P = 0.66) (**Figure 8**).

### The Protein Expression of CD133 and TTL of Patients With High-Grade Gliomas

**Figure 9:**
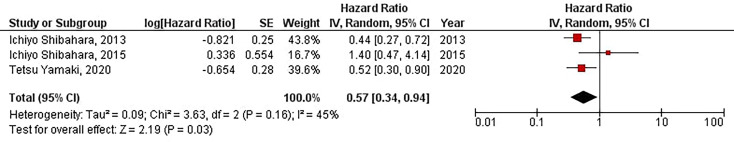
The forest plot of studies evaluating the prognostic value of CD133 in determining the TTL of patients with high-grade gliomas.

Our results have shown that increased protein expression of CD133 is significantly associated with favorable TTL in patients with high-grade gliomas (HR = 0.57, 95% CI: 0.34 – 0.94, P = 0.03). Besides, we have found no significant heterogeneity between the included studies (I^2^ = 45%, P = 0.16) (**Figure 9**).

### The Evaluation of Potential Bias in the Included Studies

We assessed the quality of included studies based on the Hayden et al. statements. Three studies have shown bias in the confounding measurement and account; however, collectively, there has been no considerable bias that can affect the obtained results (**Table 2**).

**Table 2:** The quality assessment of included studies based on the Hayden et al. checklists.

First author and the year of publication	Study participation	Study attrition	Prognostic factor measurement	Outcome measurement	Confounding measurement and account	Analysis
Tetsu Yamaki, 2020 (17)	***	***	***	***	***	***
Yasuo Iwadate, 2017 (15)	***	***	***	***	***	***
Yasuo Iwadate, 2016 (14)	***	***	***	***	***	***
Ichiyo Shibahara, 2015 (16)	***	***	**	***	***	***
Rikke H Dahlrot, 2014 (22)	***	***	***	***	*	**
Jung Ha Shin, 2013 (23)	***	***	***	***	***	***
Ichiyo Shibahara, 2013 (19)	***	***	***	***	***	***
Consolación Melguizo 2012 (24)	***	***	***	***	*	**
Kyung-Jung Kim, 2011 (25)	***	***	***	***	***	***
JIE HE, 2011 (26)	***	***	***	*	*	***
Roberto Pallini, 2008 (27)	***	***	***	***	***	***
Felix Zeppernick, 2008 (28)	***	**	***	***	***	***

***Bias might not present; **Bias might be partly present; *Bias might be present.

### Evaluating Potential Publication Bias

Based on our results, there has been no significant publication bias in our obtained results regarding the prognostic value of CD133 overexpression in patients with high-grade gliomas, the prognostic value of CD133 overexpression in patients with high-grade gliomas with 2% cut-off, the prognostic value of CD133 overexpression in patients with high-grade gliomas with 10% cut-off, and the TTD of patients with high-grade gliomas (**Figures 1S–4S**). However, our results have demonstrated significant publication bias in the studies evaluating the TTL of patients with high-grade gliomas (**Figure 5S**).

## Discussion

Although multiple clinical studies have investigated the prognostic value of CD133 in predictive tumor relapse and recurrence patterns in patients with high-grade gliomas, the published results are inconclusive. Therefore, it is necessary to clarify its prognostic value in predicting tumor relapse and recurrence patterns in patients with high-grade gliomas.

As a transmembrane glycoprotein, CD133 has been implicated in glioblastoma growth (11). In 2004, Singh et al. reported that only brain tumor cells with CD133-positive phenotype could initiate tumor development in mice brains (29). Liu et al. have indicated that CD133-positive glioblastoma cells overexpress the genes involved in inhibiting apoptosis and promoting stemness, i.e., Nestin, CD90, CD44, MGMT, CXCR4, and Musashi-1 (30). Furthermore, CD133 can confer radioresistance to glioblastoma cells and promote radiation-induced DNA damage repair (12). Besides radioresistance, CD133 upregulation has been associated with chemoresistance. Poon et al. have shown that there has been a strong association between adducin 3, a cytoskeletal factor linked with chemoresistance, with CD133. Indeed, this co-expression has been highly associated with a temozolomide-resistant state in glioblastoma cells (31). Consistent with this, Nakai et al. have reported a remarkable association between CD133 and MDR1 in glioblastoma cells and resected glioblastoma tissues, indicating the critical role of CD133 in maintaining chemoresistance (32). Therefore, CD133 can be considered a critical factor in chemo/radioresistance development in glioblastoma, which can reproduce glioma after initial therapy.

Our results have indicated that increased protein expression of CD133 is significantly associated with the inferior PFS of patients with high-grade gliomas (HR = 1.72, 95% CI: 1.22 – 2.42, P = 0.002). However, the high heterogeneity between studies has urged us to conduct meta-analyses based on the cut-offs of CD133 protein expression (I^2^ = 65%, P = 0.003) (**Figure 2**). Our results have demonstrated that with the threshold of 10%, CD133 protein overexpression is significantly associated with the inferior PFS of affected patients (HR = 1.82, 95% CI: 1.10 – 3.01, P = 0.02). Consistent with our observed results, Wang et al. have also indicated that protein/gene overexpression of CD133 is associated with the inferior PFS of patients with low-grade patients (33). Although it has been previously shown that CD133 overexpression is associated with worsened PFS of glioma patients, those results are based on pooling the protein and gene expression of CD133, which can be misleading (33). Besides, pooling data from low-grade gliomas and high-grade gliomas can also lead to misleading results (34). Moreover, without defining the cut-off for considering CD133 expression as overexpressed, the clinical translation of these kinds of studies might be at stake. In contrast, with including recently published studies and analyzing the protein expression of CD133 based on glioma grades and their defined cut-offs, the current updated study indicates that CD133 overexpression with 10% cut-off is associated with the inferior PFS in patients with high-grade gliomas.

For the first time, our meta-analysis has shown that increased protein expression of CD133 is associated with the inferior TTD of patients with glioblastoma and high-grade glioma patients (HR = 3.32, 95% CI: 1.81 – 6.07, P = 0.0001, and HR = 1.64, 95% CI: 1.18 – 2.29, P = 0.003, respectively). Indeed, our results have indicated that upregulated protein expression of CD133 is associated with sooner distant tumor relapse in glioblastoma and high-grade glioma patients. Besides, our results have demonstrated that increased protein expression of CD133 is associated with the improved TTL of glioblastoma patients (HR = 0.47, 95% CI: 0.33 – 0.68, P < 0.0001). Although our study has also indicated that elevated protein expression of CD133 is associated with favorable TTL of patients with high-grade gliomas (HR = 0.57, 95% CI: 0.35 – 0.94, P = 0.03), there has been significant publication bias among the included studies (**Figure 5S**). Thus, further investigations are required for evaluating the association between increased CD133 protein expression with the TTL of these patients. Collectively, our results have indicated that increased protein expression of CD133 is associated with sooner distant recurrence of glioblastoma and high-grade gliomas on MRI. Also, increased protein expression of CD133 is associated with improved TTL of glioblastomas on MRI. Therefore, the protein expression of CD133 can be a valuable prognostic factor for predicting the recurrence patterns of glioblastoma and high-grade gliomas in affected patients.

The current study has several limitations. First, we only included the studies that were published in English. Its second limitation stems from the nature of cohort studies that, unlike randomized clinical trials, all the confounding variables cannot be addressed. Nevertheless, the current study has several strengths as well. First, for the first time, our study has sorted out the prognostic value of protein expression of CD133, as the functional form of CD133, in predicting the relapse and recurrence pattern of high-grade gliomas based on defined cut-offs. Second, our study has linked a cancer stem cell marker and preclinical findings with clinical and imaging findings, which pave the way for more investigations to correlate the cancer stem markers with imaging findings.

## Conclusion

With a 10% cut-off, increased protein expression of CD133 is associated with the inferior PFS of patients with high-grade gliomas. Besides, the increased protein expression of CD133 is associated with sooner distant tumor recurrence and improved TTL of glioblastomas. Also, we have found that elevated protein expression of CD133 is associated with a shorter time of distant tumor recurrence in affected patients with high-grade gliomas. Overall, the protein overexpression of CD133 can be a valuable prognostic biomarker for predicting the relapse and recurrence pattern of high-grade gliomas.

## Data Availability Statement

The original contributions presented in the study are included in the article/**Supplementary Material**. Further inquiries can be directed to the corresponding authors.

## Author Contributions

All authors contributed to the article and approved the submitted version. MA has come up with the research question, selected the studies, extracted the data, analyzed the data, and wrote most of the manuscript. NH has selected the studies, interpreted the results, and provided the tables. ZA and OB have critically reviewed the paper, improved the quality of the manuscript. NS and BB have supervised the study and critically reviewed the manuscript.

## Conflict of Interest

The authors declare that the research was conducted in the absence of any commercial or financial relationships that could be construed as a potential conflict of interest.

## Publisher’s Note

All claims expressed in this article are solely those of the authors and do not necessarily represent those of their affiliated organizations, or those of the publisher, the editors and the reviewers. Any product that may be evaluated in this article, or claim that may be made by its manufacturer, is not guaranteed or endorsed by the publisher.
