# Supplementary material for: The Prognostic Value of CD133 in Predicting the Relapse and Recurrence Pattern of High-Grade Gliomas on MRI: A Meta-Analysis
Source: Front Oncol. 2021 Sep 2;11:722833. doi: 10.3389/fonc.2021.722833 (PMC8445366; doi:10.3389/fonc.2021.722833)
Supplement: Supplementary file 1 [file DataSheet_1.docx]

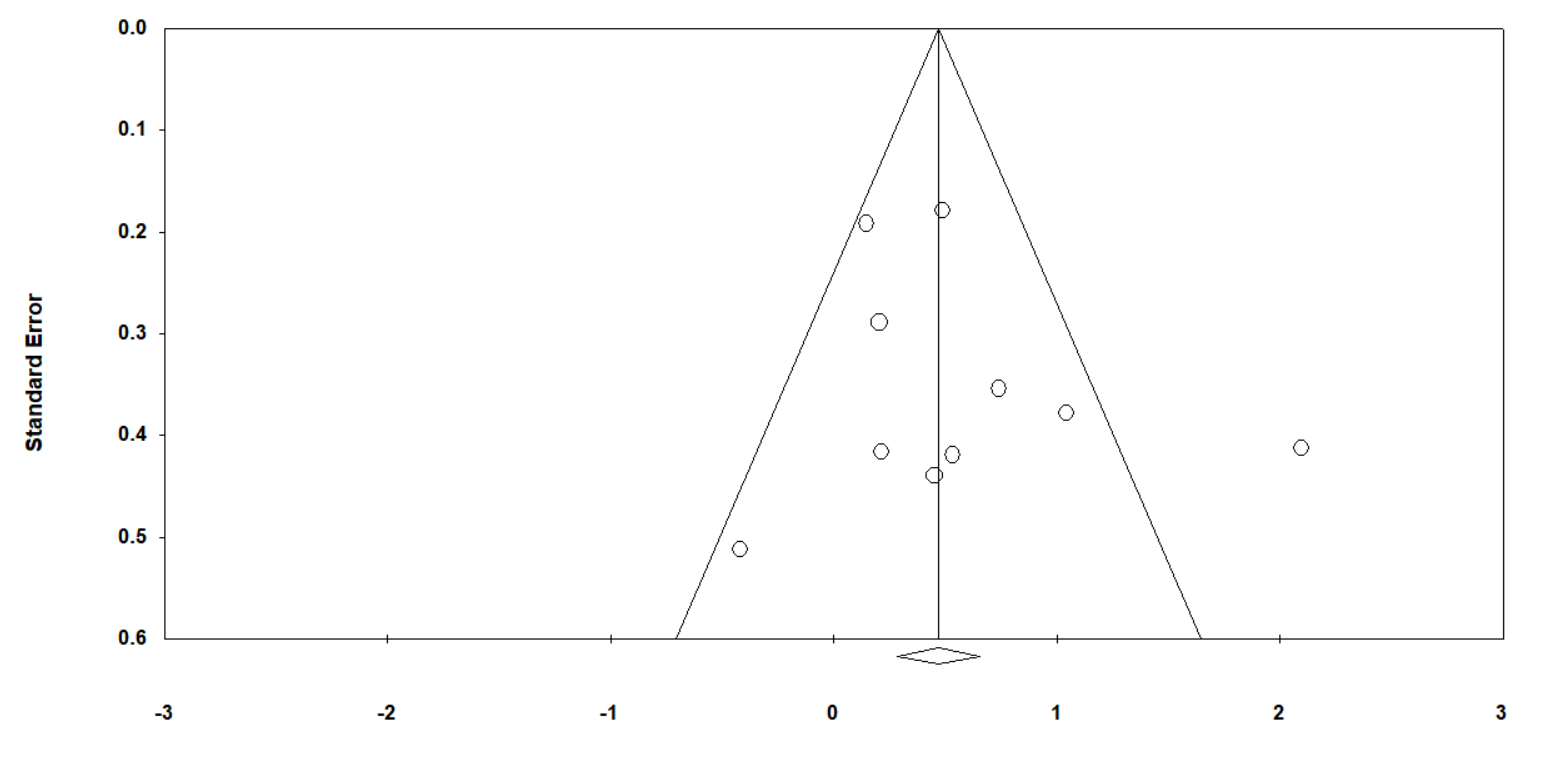


Figure 1S. The funnel plot of studies evaluating the prognostic value of CD133 overexpression in determining the PFS of patients with high-grade gliomas (Egger test P-value 1 tailed=0.22538, and P-value 2-tailed=0.45076)


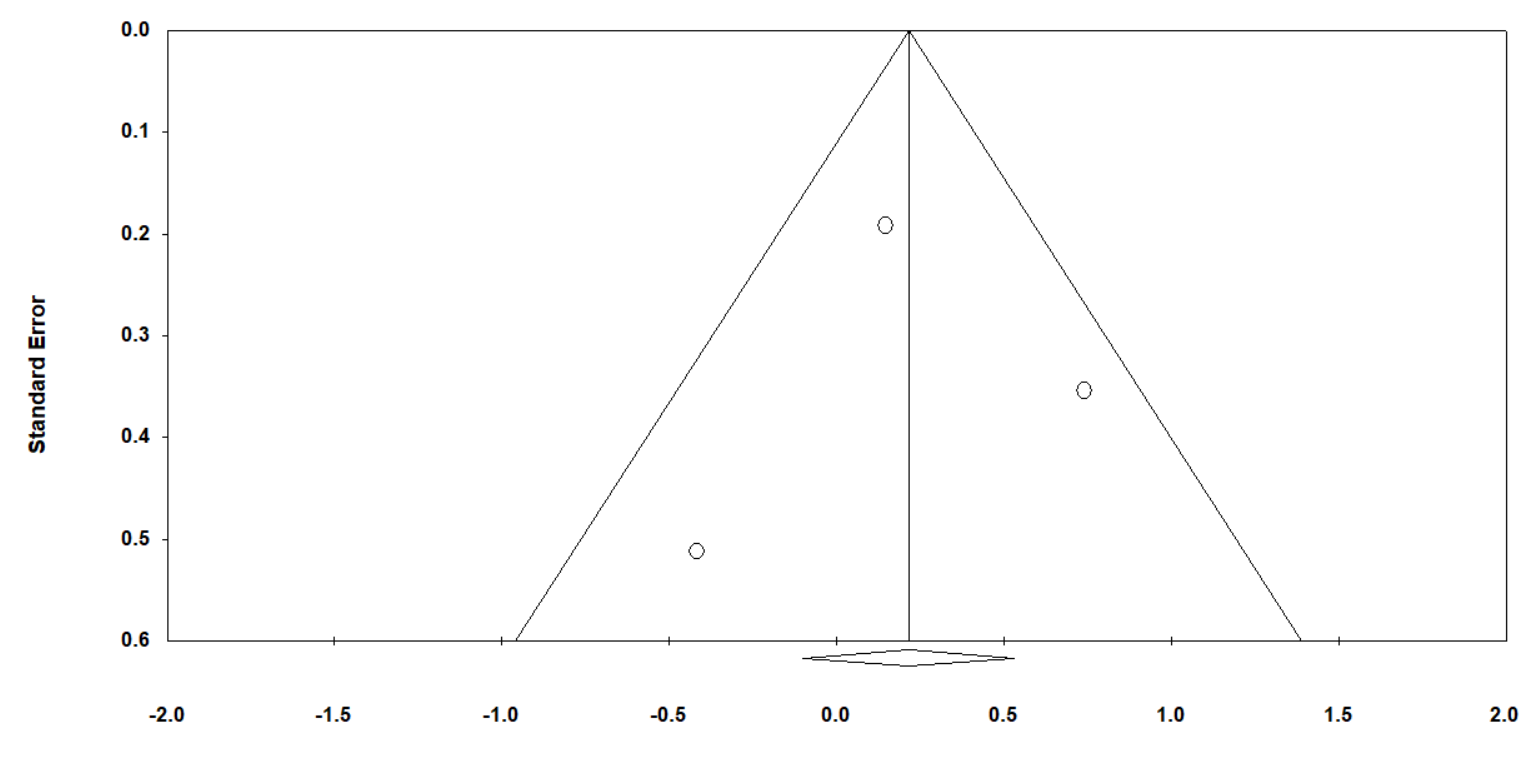


Figure 2S. The funnel plot of studies evaluating the prognostic value of CD133 overexpression with 2% cut-off in determining the PFS of patients with high-grade gliomas (Egger test P-value 1 tailed=0.47849, and P-value 2-tailed=0.95697)


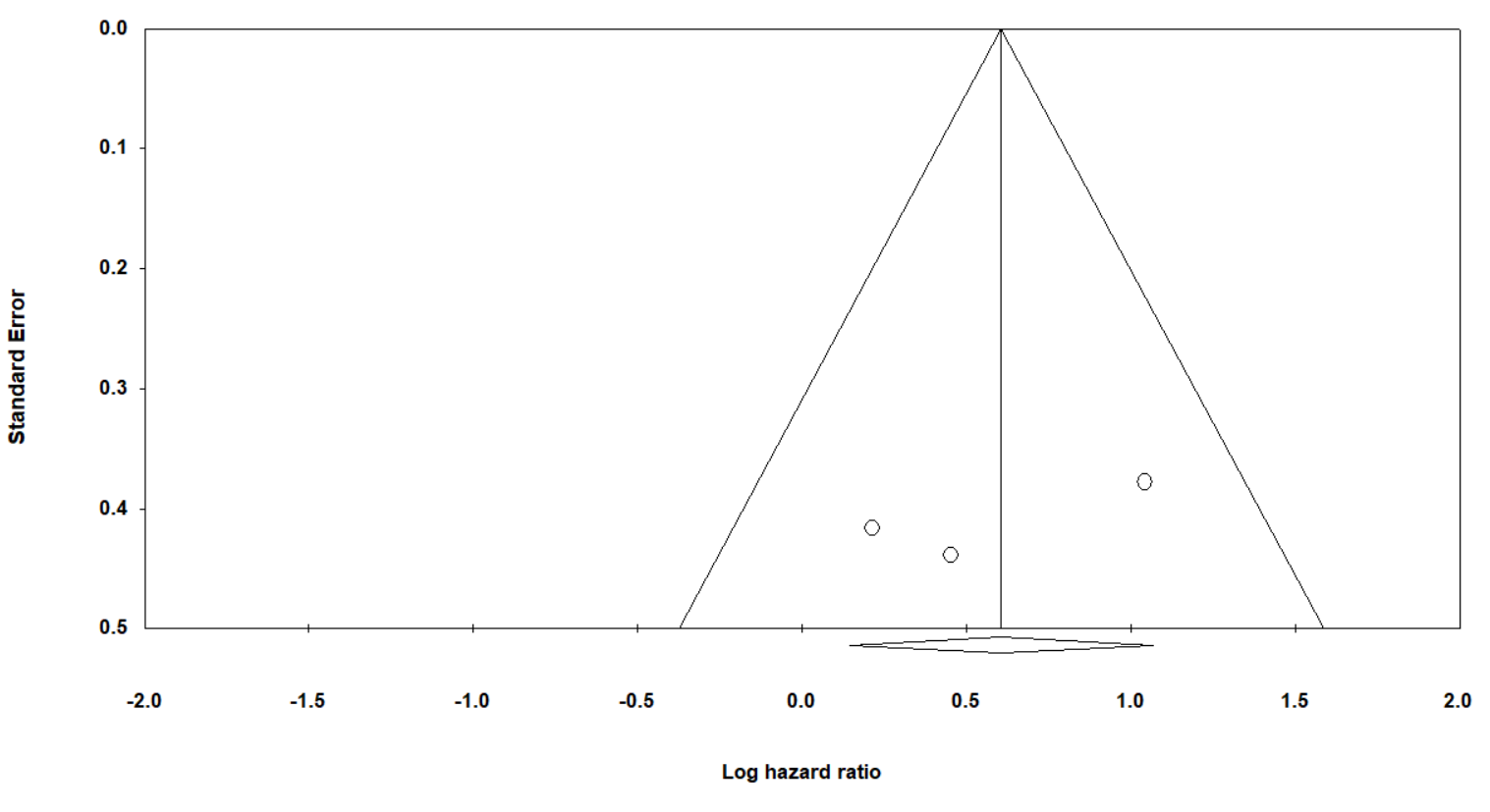


Figure 3S. The funnel plot of studies evaluating the prognostic value of CD133 overexpression with 10% cut-off in determining the PFS of patients with high-grade gliomas (Egger test P-value 1 tailed=0.19738, and P-value 2-tailed=0.39476)


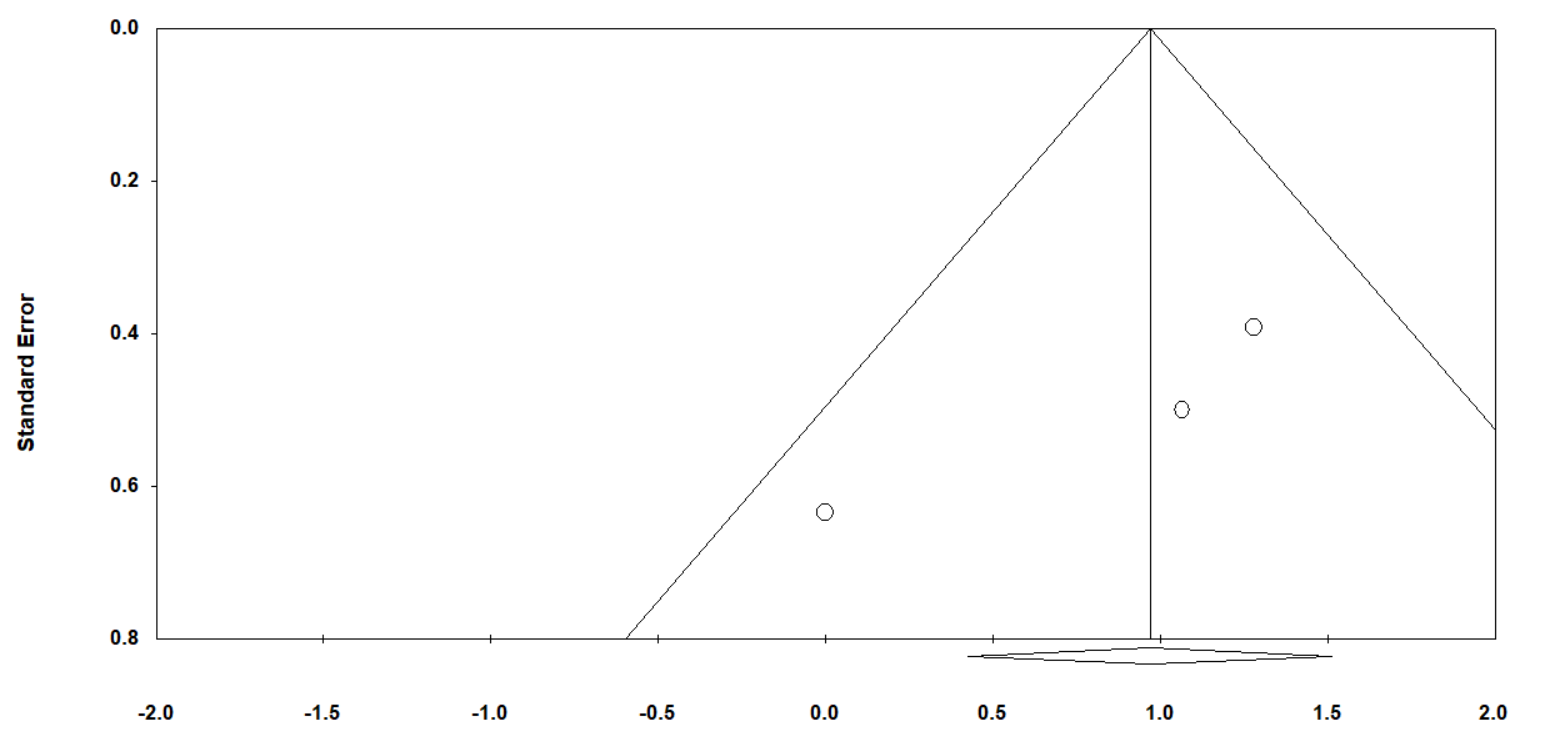


Figure 4S. The funnel plot of studies evaluating the prognostic value of CD133 in determining the TTD of patients with high-grade gliomas (Egger test P-value 1 tailed=0.10841, and P-value 2-tailed=0.21681)


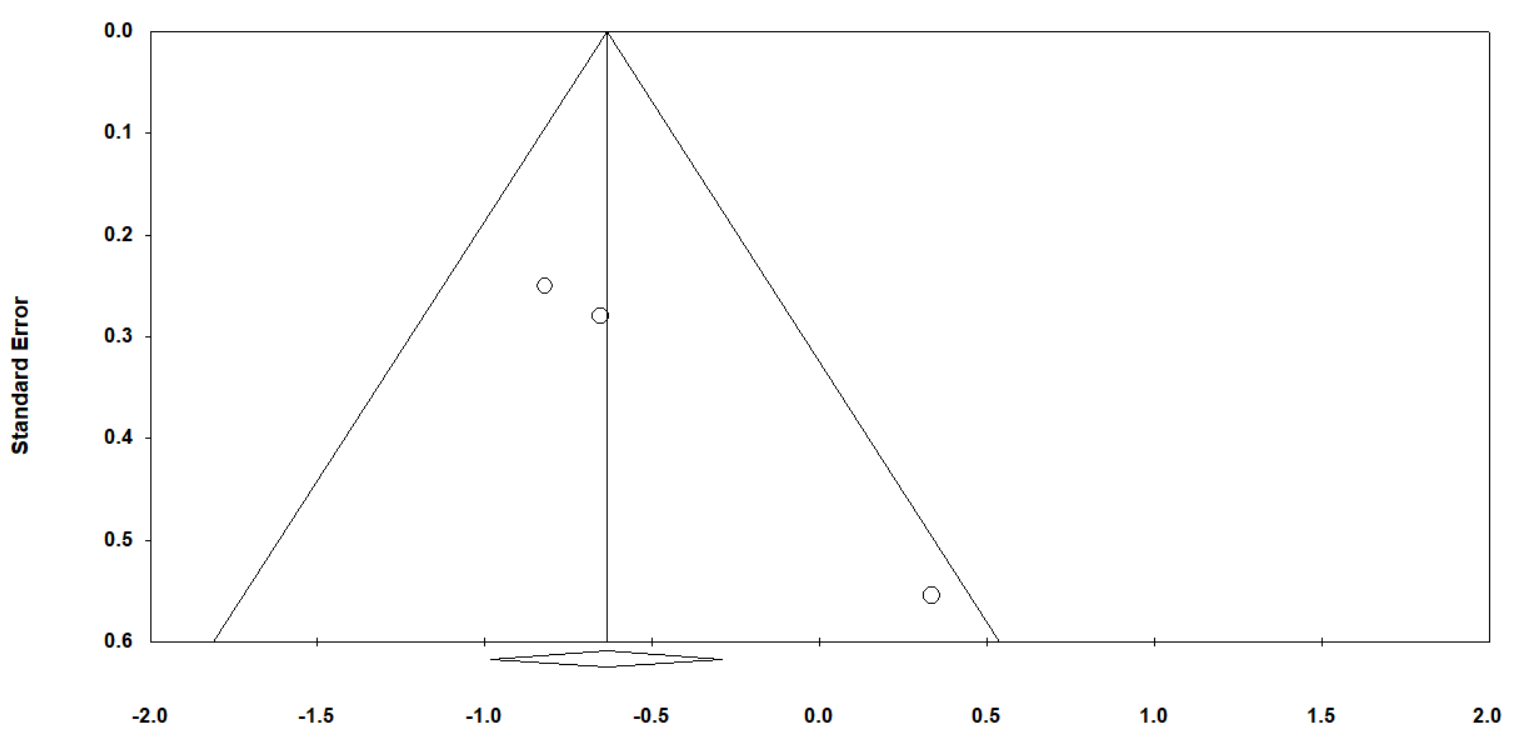


Figure 5S. The funnel plot of studies evaluating the prognostic value of CD133 in determining the TTL of patients with high-grade gliomas (Egger test P-value 1 tailed=0.02430, and P-value 2-tailed=0.04860)
